# Genome Sequence of Enterococcus faecalis NVIT04, Isolated from Nasonia vitripennis

**DOI:** 10.1128/MRA.01156-18

**Published:** 2019-01-31

**Authors:** Guan-Hong Wang, Robert M. Brucker

**Affiliations:** a The Rowland Institute at Harvard University, Harvard University, Cambridge, Massachusetts, USA; Georgia Institute of Technology

## Abstract

Enterococcus faecalis is a Gram-positive, lactic acid-producing coccus which can be found as a member of the gut microbiome in many animal species and is a potential pathogen in humans. Here, we describe the genome sequence of an E. faecalis strain isolated from the gut microbiome of the hymenopteran model Nasonia vitripennis.

## ANNOUNCEMENT

The *Enterococcus* genus is a consistent member of the mammalian, avian, reptilian, and insect gut microbiome and has been implicated as both a potential pathogen (1, 2) and a beneficial microorganism (3, 4). The species Enterococcus faecalis is typically classified as an opportunistic pathogen, especially in immunocompromised patients. While many strains have been reported to possess natural antibiotic resistance, there has been an increasing number of multidrug-resistant strains of the bacterium emerging since the 1970s. This surge of multidrug-resistant strains drives the need for further understanding of the genetic diversity and natural reservoirs of the genus (1).

E. faecalis has been detected in relatively low abundance in the gut microbiome of the parasitic wasp genus *Nasonia* (∼2%) (5), a well-established model organism for the host-symbiont interaction (5, 6). However, the potential role of *Enterococcus* species in the health and development of the wasp host is currently unknown. We sought to assess the potential function of E. faecalis within the microbiome of N. vitripennis strain AsymCx. After surface sterilizing whole *Nasonia* animals with 10% bleach for 2 min and then homogenizing them in sterile phosphate-buffered saline (PBS), we then plated the 1× aliquots of the homogenate onto several bacterial agar medium types. Specifically, we isolated E. faecalis using bile esculin agar (catalog no. 48300; Sigma-Aldrich) as a selective medium and confirmed the taxonomic identity of a resulting colony by Sanger sequencing of the 16S rRNA gene before performing whole-genome sequencing.

Bacterial genomic DNA was isolated from E. faecalis NVIT04 cells cultured in 100 ml of nutrition broth (catalog no. 1.05443.0500; Sigma-Aldrich) medium at 30°C and 250 rpm for 18 h in a flask. The culture was centrifuged at 6,000 rpm for 5 min, and the resulting cell pellet was used for genome extraction using the DNeasy blood and tissue kit (Qiagen, Hilden, Germany). DNA was quantified using the double-stranded DNA (dsDNA) high-sensitivity (HS) assay kit on the Qubit 2.0 fluorometer (Life Technologies, Waltham, MA, USA).

Library preparation and genome sequencing were performed at MicrobesNG (Birmingham, United Kingdom). The genomic DNA library was prepared using the Nextera XT library prep kit (Illumina), following the manufacturer’s protocol, with a Microlab STAR automated liquid-handling system (Hamilton). The library was sequenced on the Illumina HiSeq platform using a 250-bp paired-end protocol.

Sequencing resulted in 727,126 reads that were trimmed using Trimmomatic 0.36 (7), and the quality was assessed using in-house scripts (MicrobesNG, Birmingham, United Kingdom) combined with SAMtools (8), BedTools (9), and the Burrows-Wheeler Aligner MEM algorithm (BWA-MEM) (10). Contig assembly was carried out with SPAdes (version 3.8) (11) via MicrobesNG. This resulted in a draft genome consisting of 27 contigs, with an *N*_50_ value of 1,478,411 bp and a total of 2,773,768 nucleotides, resulting in approximately 110-fold coverage.

The genome of E. faecalis NVIT04 shared 99% nucleotide identity with available reference genomes of E. faecalis strains sorialis (GenBank accession no. CP015883), V583 (GenBank accession no. AE016830) (12), and DD14 (GenBank accession no. CP021161). The genome of E. faecalis NVIT04 consists presumably of a single chromosome (2,765,246 bp) and exhibits a G+C content of 37.46%. No plasmids were detected based on analysis with the PlasmidFinder (version 1.3) (13). Annotation was performed using Prokka 1.12 (14). The genome encodes 2,561 predicted protein-encoding genes with known functions and 794 (31.00%) coding for hypothetical proteins. The draft genome encodes 5 rRNAs and 54 tRNAs identified using RNAmmer (15) and tRNAscan (16), respectively. All the software programs mentioned above were used with default settings.

Four putative antibiotic resistance genes were identified using the CosmosID package (version August 2018; CosmosID, Inc., Rockville, MD, USA), namely, a multidrug resistance efflux pump gene (*emeA*), an efflux pump gene (*isaA*), a dihydrofolate reductase gene (*dfrE*), and a macrolide resistance gene (*mphD*). Likewise, 18 virulence factors were identified using the CosmosID package, namely, a collagen adhesion gene (*ace*), pheromoneprecursor lipoprotein genes (*cad, camE, cCF10*, and *cOB1*), endocarditis- and biofilm-associated pilus genes (*ebpA* and *ebpC*), an endocarditis antigen A gene (*efaAfs*), a gene encoding enterococcal leucine-rich internalin-like protein A (*elrA*), a gelatinase gene (gelE), a hyaluronidase gene (*hylA*), a sortase A gene (*srtA*), a thiol peroxidase gene (*tpx*), FsrABDC signal transduction system genes (*fsrA, fsrB,* and *fsrC*), a gene encoding the regulation of malate uptake (*malR*), and a serine protease (*sprE*). The large number of antibiotic resistance genes and virulence factors identified in the E. faecalis NVIT04 genome analysis indicates the potential of isolates to be pathogenic and supports the diverse E. faecalis-host relationships across animal species.

### Data availability.

This whole-genome shotgun project has been deposited at DDBJ/ENA/GenBank under the accession no. QUAG00000000. The version described in this paper is the first version, QUAG01000000, and the GenInfo identifier (GI) no. is 1467817911 2. The BioProject accession no. is PRJNA484798 for the SRA data.

## References

[1] Gilmore MS, Clewell DB, Ike Y, Shankar N (ed). 2014 Enterococci: from commensals to leading causes of drug resistant infection. Massachusetts Eye and Ear Infirmary, Boston, MA. 24649510

[2] Steck N, Hoffmann M, Sava IG, Kim SC, Hahne H, Tonkonogy SL, Mair K, Krueger D, Pruteanu M, Shanahan F, Vogelmann R, Schemann M, Kuster B, Sartor RB, Haller D 2011 *Enterococcus faecalis* metalloprotease compromises epithelial barrier and contributes to intestinal inflammation. Gastroenterology 141:959–971. doi:10.1053/j.gastro.2011.05.035. 21699778

[3] Cebrián R, Baños A, Valdivia E, Pérez-Pulido R, Martínez-Bueno M, Maqueda M 2012 Characterization of functional, safety, and probiotic properties of *Enterococcus faecalis* UGRA10, a new AS-48-producer strain. Food Microbiol 30:59–67. doi:10.1016/j.fm.2011.12.002. 22265284

[4] Soerjadi AS, Lloyd AB, Cumming RB 1978 *Streptococcus faecalis*, a bacterial isolate which protects young chickens from enteric invasion by Salmonellae. Aust Vet J 54:549–550. 111659 10.1111/j.1751-0813.1978.tb00337.x

[5] Brucker RM, Bordenstein SR 2012 The roles of host evolutionary relationships (genus: *Nasonia*) and development in structuring microbial communities. Evolution 66:349–362. doi:10.1111/j.1558-5646.2011.01454.x. 22276533

[6] Brucker RM, Bordenstein SR 2013 The hologenomic basis of speciation: gut bacteria cause hybrid lethality in the genus *Nasonia*. Science 341:667–669. doi:10.1126/science.1240659. 23868918

[7] Bolger AM, Lohse M, Usadel B 2014 Trimmomatic: a flexible trimmer for Illumina sequence data. Bioinformatics 30:2114–2120. doi:10.1093/bioinformatics/btu170. 24695404 PMC4103590

[8] Li H 2011 A statistical framework for SNP calling, mutation discovery, association mapping and population genetical parameter estimation from sequencing data. Bioinformatics 27:2987–2993. doi:10.1093/bioinformatics/btr509. 21903627 PMC3198575

[9] Quinlan AR 2014 BEDTools: the Swiss-Army tool for genome feature analysis. Curr Protoc Bioinformatics 47:11 12 1–34. doi:10.1002/0471250953.bi1112s47. PMC4213956 25199790

[10] Li H, Durbin R 2009 Fast and accurate short read alignment with Burrows–Wheeler transform. Bioinformatics 25:1754–1760. doi:10.1093/bioinformatics/btp324. 19451168 PMC2705234

[11] Bankevich A, Nurk S, Antipov D, Gurevich AA, Dvorkin M, Kulikov AS, Lesin VM, Nikolenko SI, Pham S, Prjibelski AD, Pyshkin AV, Sirotkin AV, Vyahhi N, Tesler G, Alekseyev MA, Pevzner PA 2012 SPAdes: a new genome assembly algorithm and its applications to single-cell sequencing. J Comput Biol 19:455–477. doi:10.1089/cmb.2012.0021. 22506599 PMC3342519

[12] Paulsen IT, Banerjei L, Myers GS, Nelson KE, Seshadri R, Read TD, Fouts DE, Eisen JA, Gill SR, Heidelberg JF, Tettelin H, Dodson RJ, Umayam L, Brinkac L, Beanan M, Daugherty S, DeBoy RT, Durkin S, Kolonay J, Madupu R, Nelson W, Vamathevan J, Tran B, Upton J, Hansen T, Shetty J, Khouri H, Utterback T, Radune D, Ketchum KA, Dougherty BA, Fraser CM 2003 Role of mobile DNA in the evolution of vancomycin-resistant *Enterococcus faecalis*. Science 299:2071–2074. doi:10.1126/science.1080613. 12663927

[13] Carattoli A, Zankari E, Garcia-Fernandez A, Larsen MV, Lund O, Villa L, Aarestrup FM, Hasman H 2014 In silico detection and typing of plasmids using PlasmidFinder and plasmid multilocus sequence typing. Antimicrob Agents Chemother 58:3895–3903. doi:10.1128/AAC.02412-14. 24777092 PMC4068535

[14] Seemann T 2014 Prokka: rapid prokaryotic genome annotation. Bioinformatics 30:2068–2069. doi:10.1093/bioinformatics/btu153. 24642063

[15] Lagesen K, Hallin P, Rodland EA, Staerfeldt HH, Rognes T, Ussery DW 2007 RNAmmer: consistent and rapid annotation of ribosomal RNA genes. Nucleic Acids Res 35:3100–3108. doi:10.1093/nar/gkm160. 17452365 PMC1888812

[16] Lowe TM, Eddy SR 1997 tRNAscan-SE: a program for improved detection of transfer RNA genes in genomic sequence. Nucleic Acids Res 25:955–964. 9023104 10.1093/nar/25.5.955 PMC146525

